# MicroRNA-148b enhances the radiosensitivity of B-cell lymphoma cells by targeting Bcl-w to promote apoptosis

**DOI:** 10.7150/ijbs.40756

**Published:** 2020-01-23

**Authors:** Si-hong Liu, Pei-pei Wang, Cun-te Chen, Dan Li, Qiong-yao Liu, Lin Lv, Xia Liu, Li-na Wang, Bao-xiu Li, Cheng-yin Weng, Xi-sheng Fang, Xiao-fei Cao, Hai-bo Mao, Xiao-jun Chen, Shao-li Luo, Shu-xiang Zheng, Guo-long Liu, Yong Wu

**Affiliations:** 1 Department of Orthopaedics, Guangzhou First People's Hospital, School of Medicine, South China University of Technology, Guangzhou, Guangdong, China; 2 Department of Oncology, Guangzhou First People's Hospital, School of Medicine, South China University of Technology, Guangzhou, Guangdong, China; 3 Department of Hematology, Guangzhou First People's Hospital, School of Medicine, South China University of Technology, Guangzhou, Guangdong, China; 4 Department of Gerontology, Guangzhou First People's Hospital, School of Medicine, South China University of Technology, Guangzhou, Guangdong, China; 5 Department of Obstetrics, Guangzhou First People's Hospital, School of Medicine, South China University of Technology, Guangzhou, Guangdong, China; 6 Guangzhou First People's Hospital, Guangzhou Medical University, Guangzhou, Guangdong, China

**Keywords:** miR-148b, radiosensitivity, B-cell lymphoma, Bcl-w, apoptosis

## Abstract

Lymphoma is a malignant disease of the hematopoietic system that typically affects B cells. The up-regulation of miR-148b is associated with radiosensitization in B-cell lymphoma (BCL). This study aimed to explore the role of miR-148b in regulating the radiosensitivity of BCL cells and to investigate the underlying mechanism. miR-148b directly targeted Bcl-w, decreased the cell viability and colony formation, while promoted apoptosis, in irradiated BCL cells. These changes were accompanied by decreased mitochondrial membrane potential, release of cytochrome C, increased levels of the cleaved caspase 9 and caspase 3, and increased expression of other proteins related to the mitochondrial apoptosis pathway. These effects of miR-148b were effectively inhibited by Bcl-w. In addition, miR-148b inhibited the growth of tumors in nude mice implanted with xenografts of irradiated Raji cells. In patients with BCL, levels of miR-148b were downregulated, while levels of Bcl-w were upregulated; a significant negative correlation between levels of miR-148b and Bcl-w was confirmed. Taken together, these experiments showed that miR-148b promoted radiation-induced apoptosis in BCL cells by targeting anti-apoptotic Bcl-w. miR-148b might be used as a marker to predict the radiosensitivity of BCL.

## Introduction

Abnormalities in apoptotic signaling contribute to tumorigenesis and tumor development; they also play a key role in radiosensitivity. Altered expression of apoptosis-related proteins such as surviving 1, BIRC7 2, Mcl-1 3, and Bcl-2 4 has been found in malignant lymphoma. Anti-apoptotic proteins in the Bcl-2 family, such as Bcl-2 5, 6 and Bcl-xl 7, 8, are key to cell survival and often overexpressed in malignant tumors, leading to increased cancer cell survival. Bcl-w is a member of the Bcl-2 family, which acts directly on pro-apoptotic proteins to inhibit apoptosis 9-11, and play a key role in the development and progression of various malignancies, and is involved in radiation-induced cell death 12. Upregulation of Bcl-w promotes tumorigenesis while downregulation of Bcl-w promotes cancer cell apoptosis 13, 14. Bcl-w may act through MYC to regulate the transcription of specific miRNA in order to increase B cell apoptosis 13. Conversely, the use of miRNA to suppress the expression of Bcl-w decreases the proliferation and invasion of clear-cell renal cell carcinoma cells 15. Cyramza (ramucirumab) is a human VEGF receptor-2 antagonist that inhibits angiogenesis and growth. The drug was approved for the treatment of multiple malignancies in 2014. Cyramza may act on Bcl-w to induce the apoptosis of HCC4006 cells 16. These findings support a role for Bcl-w in B-cell lymphoma (BCL).

MiRNAs are critical to cell growth, development, proliferation, differentiation, and death 17. Growing evidence has demonstrated that miRNAs are involved in the regulation of tumor radiosensitivity 18-27. It has previously been reported that miR-148b acts as a tumor suppressor in the context of lung cancer 28, cervical cancer 29, and gastric cancer 30. In addition, miR-148b is downregulated in breast cancer 31, colorectal cancer 32, and hepatocellular carcinoma 2. We have reported that radiation therapy upregulates miR-148b, thus enhancing the radiosensitivity of BCL cells by promoting radiation-induced apoptosis 33.

Overexpression of miR-148a in a colorectal cancer cell line led to decreased expression of Bcl-2 and Bcl-XL 34. Bcl-w protein is an important anti-apoptotic protein in the Bcl-2 family, which regulates the mitochondrial apoptotic pathway 35. Bcl-w knockout animals showed significantly more apoptosis than wild-type counterparts in small intestine as well as mid-colon after irradiation 36. Bioinformatics analysis and the results of preliminary experiments indicate that Bcl-w may be the target of miR-148b 35. However, it is not clear if miR-148b really acts on Bcl-w and plays a role in the radiosensitivity of BCL.

In this study, we sought to confirm Bcl-w as the target gene of miR-148b in BCL cells and to investigate the role of miR-148b and Bcl-w in the regulation of radiosensitivity and mitochondrial apoptosis in BCL cells.

## Materials and Methods

### Patients

A total of 51 patients diagnosed with BCL based on World Health Organization (WHO) classification criteria were enrolled at our hospital during the period from February 2017 to March 2019. Peripheral blood specimens from 21 newly diagnosed patients were collected before treatment. Peripheral blood specimens from 18 healthy volunteers were included as control. Lymphatic tissue specimens from 30 newly diagnosed patients were collected before treatment. Lymphatic tissue specimens from 18 healthy volunteers were included as control (Table 1). All subjects gave their informed consent for inclusion before they participated in the study. The study was conducted in accordance with the Declaration of Helsinki, and the protocol was approved by the Ethics Committee of Guangzhou First People's Hospital (Project identification code: K-2017-133-01).

### Isolation of mononuclear cells

A peripheral blood sample was added to the surface of 5 ml Ficoll (1.077g/mL; GE Healthcare Life Sciences), then centrifuged at 670×*g* in a centrifuge at 25℃ for 25 minutes. After centrifugation, the liquid was divided into three layers. The narrow white turbid layer between the upper and middle layers, which consisted mainly of mononuclear cells (MNCs), was pipetted into another centrifuge tube, and MNCs were washed twice with PBS. Finally, 5-10 × 10^6^ MNCs were stored in TRIzol reagent (Invitrogen).

### Cell culture

Raji and SU-DHL-10 human BCL cell lines were obtained from ATCC and cultured in RPMI-1640 medium (Hyclone, USA) containing 10% (v/v) fetal bovine serum (Gibco, USA), 100 U/ml penicillin, and 100μg/ml streptomycin (Gibco, USA) in an incubator containing 5% CO_2_ at 37˚C. All experiments were performed with exponentially growing cells. HEK-293T cells were obtained from the Chinese Academy of Sciences and cultured in Dulbecco modified Eagle medium containing 10% (v/v) fetal bovine serum (Gibco, USA), 100 mg/mL penicillin, and 100 U/mL streptomycin (Gibco, USA) in an incubator containing 5% CO_2_ at 37˚C.

### Irradiation

External beam radiation was performed by using an Elekta Precise Linear Accelerator (Elekta Oncology Systems, UK), equipped with a 6-MV photon beam. A field size of 40×40 cm was used. Petri dishes were placed in a 1.5-cm superflab bolus, at a distance of 100 cm from the source. The calculated monitoring unit (MU) delivered the dose to a depth of d_max_ at 2.5Gy/min. Cells were removed from the incubator and transferred to the site for radiation. The radiation dose of 2 Gy or 4 Gy was verified and confirmed after calibration with the accelerator's dosimeter. The blank or vector-transfected cells after irradiation were used as controls.

### Luciferase reporter assay

The wild type 3'UTR sequence of Bcl-w (wt 3 'UTR), which contains the putative miR-148b binding site, was amplified by PCR using the Bcl-w wt primer pair (Table 2). A mutated 3' UTR (mut 3' UTR) of Bcl-w was generated through site-directed mutagenesis with Bcl-w mut primer pair (Table 2) using a Quik-Change Site-Directed Mutagenesis Kit (Stratagene, USA). Both Bcl-w wt 3' UTR and Bcl-w mut 3' UTR were fused with the luciferase reporter gene in the psiCHECK-2 vector (Promega). Raji cells and SU-DHL-10 cells were divided into four groups. One group was co-transfected with Wt 3'UTR vectors, control vectors of psiCHECK-2 (Promega, USA) encoding Renilla luciferase and miR-148b mimic; one group was co-transfected with Wt 3'UTR vectors, control vectors of psiCHECK-2 encoding Renilla luciferase and miR-control; one group was co-transfected with mut 3'UTR vectors, control vectors of psiCHECK-2, and miR-control; and the fourth group was co-transfected with mut 3'UTR vectors, and a control vector encoding Renilla luciferase, control vectors of psiCHECK-2 (Promega, USA) and miR-control, with Lipofectamine 2000 (Invitrogen). After 48h, levels of luciferase activity were detected using the Dual-Luciferase Reporter Assay System (Promega) and normalized with the Renilla values. Values are presented as the ratio of firefly/Renilla values.

### Construction of miR-148b mimic and inhibitor lentivirus vectors

A fragment encoding the miR-148b sequence was amplified from human genomic DNA by PCR, then cloned into the BamHI/XhoI sites of pLVX-IRES-Neo vector to make lentivirus vector pLVX-IRES-Neo-miR-148b (miR-148b mimic). A fragment encoding the miR-148b inhibitor sequence was amplified by PCR, then cloned into the BamHI/EcoRI sites of pLVX-shRNA2 vector to create miR-148b inhibitor.

### Lentivirus production and infection

First, 5×10^6^ 239T cells were cultured overnight in a 100-mm plastic petri dish. Second, 293T cells were transfected with miR-148b mimic or inhibitor lentivirus using pLVX-IRES-Neo-miR-148b or pLVX-shRNA2+miR-148b-inhibitor, pHelper 1.0, and pHelper 2.0 in combination with Lipofectamine 2000 (Invitrogen). After 24 hours, the aforementioned medium was collected, centrifuged at 3,000 rpm for 10 minutes at 4˚C, then filtered using a 0.45-mm filter and the filtrate containing the lentivirus was collected for infection. Then 80% to 90% confluent Raji cells and SU-DHL-10 cells were infected with miR-148b mimic or inhibitor in the presence of 10 mg/mL hexadimethrine bromide (Sigma-Aldrich, St. Louis, MO, USA). After 24 hours, a fluorescence activated cell sorter (Becton Dickinson, Mountain View, CA) was used to measure the expression of green fluorescent protein (GFP), as an index for infection efficiency.

### Cell transfection

The open reading frame (ORF) of Bcl-w was amplified and cloned into pCDNA3.1. Bcl-w small interfering RNA (siRNA) was obtained from Sigma (USA). Raji cells and SU-DHL-10 cells were transfected with Bcl-w expression vector, empty plasmid, or Bcl-w siRNA with Lipofectamine 2000 (Invitrogen).

### Quantitative real-time PCR analysis

Expression levels of miR-148b were determined using the miRNA-specific assay kit (Takara, China), and U6 was used as an internal control. Expression levels of Bcl-w were detected using SYBR Premix Ex Taq II (Takara, China), with β-actin as an internal control. Quantitative real-time PCR (qRT-PCR) were performed on an ABI PRISM® 7500 Sequence Detection System. The signals were normalized to the internal control and expressed as 2^-ΔΔCt^.

### Immunohistochemistry and scoring

Pathological lymph tissue specimens from 30 newly diagnosed BCL patients were paraffin-embedded and cut into 2μm sections. Immunostaining was performed as previously described 37. Briefly, slides were deparaffinized, rehydrated in a series of degrading ethanol solutions and boiled in EDTA (1 mM, pH 8.0). After the endogenous peroxidase was quenched with 0.3% H_2_O_2_, slides were incubated with the primary antibody of mouse anti-human Bcl-w (Abcam; 1:200) overnight. After incubation with the HRP-conjugated secondary antibody (DAKO EnVision^TM^ Detection Kit), the signal was visualized using the A solution in the DAKO kit. Sections were then counterstained with hematoxylin. The number of positively stained cells and the staining intensity of 5 randomly selected fields of view were manually counted at 400x high-power magnification (cells/HPF). The score was then determined by calculating the average number of positively stained cells per HPF.

### Histological analysis of TUNEL staining

Pathological lymphatic tissue from 30 newly diagnosed BCL patients was paraffin-embedded and cut into 2μm sections. The slides were deparaffinized, rehydrated in a series of degrading ethanol solutions and then rinsed with 0.1% Triton X-100 for cell penetration. Apoptotic lymph tissue cells were detected by terminal deoxynucleotidyl transferase-mediated dUTP nick-end labeling (TUNEL) using the TransDetectIn Situ Fluorescein TUNEL Cell Apoptosis Detection Kit (Transgen Biotech, China) 38. The apoptotic index was determined by TUNEL assay as the percentage of apoptotic events per lymphocyte population in five random fields at 400x high-power magnification (cells/HPF).

### Cell viability assay

Cells were seeded at a density of 1×10^4^ cells/well into 96-well plates. After irradiation, cells were cultured in a 5% CO_2_ chamber at 37˚C. Viable cells were evaluated using the CCK-8 Assay kit (Beyotime, China) according to the manufacturer's instructions over four consecutive days. CCK-8 was added to the 96-well plates, which were incubated at 37˚C for 4 hours. The optical density (OD) value of each well was read at a wavelength of 450 nm using a microplate reader (multiscan MK3, Thermo Fisher Scientific).

### Clonogenic assay

Cells were seeded at a density of 100 cells/ 300 µl complete medium and Matrigel (BD Biosciences, USA) into each well of 96-well plates. They were incubated in a chamber containing 5% CO_2_ at 37˚C, and then stained with Crystal Violet on Day 8. Colonies were counted with the AID iSpot Reader System.

### Annexin V-propidium iodide apoptosis assay

Approximately 5×10^5^ cells were incubated with Alexa Fluor 488-conjugated annexin V and propidium iodide (PI; Keygen Biotech, China). Apoptotic cells were detected using a FACSCalibur flow cytometer (BD Biosciences, USA) with annexin V+/ PI+ cells labeled as necrotic cells and annexin V+/ PI- cells labeled as apoptotic cells.

### Mitochondrial membrane permeability

Mitochondrial membrane potential (MMP) was assessed using fluorescent probe JC-1 (Life Technologies). Cells seeded in 6-well plates were treated with JC-1 (1 mL per well) at a concentration of 10 ug/mL for 20 minutes at 37 ºC. Fluorescence intensity was detected by a FACSCalibur flow cytometer (BD Biosciences, USA) immediately after washing with serum-free medium. For JC-1 green, Ex = 514 nm and Em = 529 nm; for JC-1 red, Ex = 585 nm and Em = 590 nm.

### Immunofluorescence analysis

Cytochrome C was examined by immunostaining with cytochrome C antibody. Briefly, cells were fixed in 4% paraformaldehyde and incubated overnight with rabbit anti-cytochrome C antibody (Abcam; 1:100), then incubated with Alexa Fluor 488-labeled goat anti-rabbit IgG antibody (Life Technologies; 1:200) for 1 hour. Nuclei were stained with DAPI. Images were observed under a laser scanning confocal microscope (Zeiss, Germany).

### Western blot analysis

Protein lysates were separated by SDS-PAGE in a 10% polyacrimide gel, then transferred to a polyvinylidene difluoride membrane (Millipore, USA). Membranes were incubated with antibody against human Bcl-w (Abcam; 1:200), Apaf-1 (Abcam; 1:500), cleaved Caspase-9 (Novus Bio; 1:1,000), cleaved Caspase-3 (CST; 1:1,000), Bax (Abcam; 1:2,000); or GAPDH (KangChen Bio-tech Inc., China). Peroxidase-conjugated goat anti-mouse IgG (H+L) (Southern Biotech) was used as the secondary antibody. The intensity of staining was visualized using an X-ray image film processor (Kodak, Japan).

### Tumorigenicity

Male BALB/c nude mice were obtained from the Laboratory Animals Center of Sun Yat-Sen University. Mice were housed under specific pathogen-free conditions in the animal facilities of South China University of Technology. Mice at the age of 6 to 8 weeks were randomly divided into four groups, each of which included 3 mice. Irradiated Raji cells (1×10^7^ in 0.2 ml of PBS/mouse) were subcutaneously inoculated into the right flank of each nude mouse included in the study. Tumor volume was calculated using the following formula: (L×W^2^)/2, with length (L) and width (W) measured every 3 days. Forty-seven days after implantation the last measurement of tumor volume was performed, and mice were euthanized by inhalation of carbon dioxide followed by cervical dislocation. All animal protocols were approved by the ethics committee at South China University of Technology.

### Statistical analysis

Each experiment was repeated at least 3 times. The difference in quantitative data between the two groups was compared using independent-sample Student 's t-test and Mann-Whitney rank-sum test, as appropriate (Table 1). Differences between multiple groups of quantitative data was performed using one-way ANOVA and Fisher 's exact test, as appropriate. The qualitative data was compared by using Chi-square test. All statistical analyses were conducted using SigmaStat 3.5 (Systat Software, San Jose, CA, USA). *P* < 0.05 was considered statistically significant.

## Results

### Bcl-w is a target of miR-148b in BCL cells

**Figure 1:**
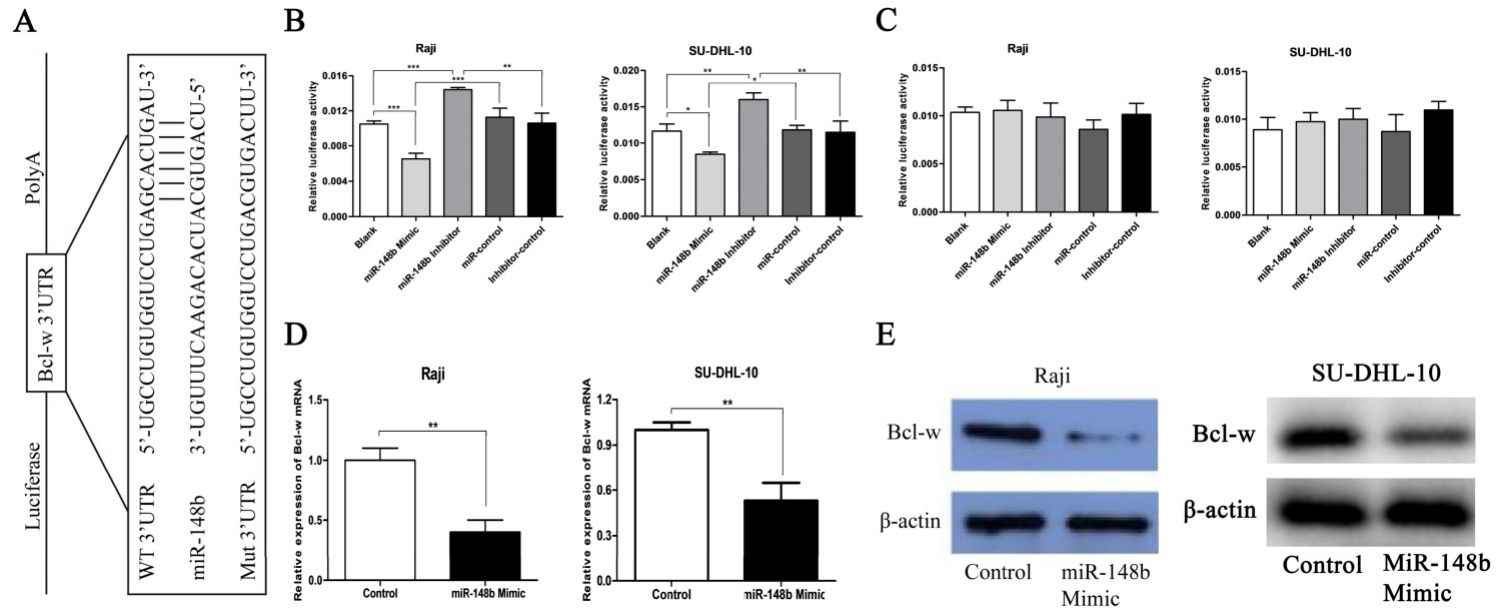
Bcl-w is a direct target of miR-148b in BCL cells. **(A)** Schematic diagram of the reporter constructs containing the predicted miR-148b binding site in the 3'UTR of Bcl-w. **(B)** miR-148b mimic or inhibitor significantly changed the luciferase activity of Bcl-w 3'UTR in Raji and SU-DHL-10 cells co-transfected with wt 3'UTR. **(C)** miR-148b mimic or inhibitor had no significant effect on the luciferase activity of mut 3'UTR in Raji and SU-DHL-10 cells co-transfected with mut 3'UTR. **(D)** PCR analysis of Bcl-w mRNA level in Raji cells and SU-DHL-10 cells transfected with control or miR-148b mimic. **(E)** Representative blots showing Bcl-w protein level in Raji cells and SU-DHL-10 cells transfected with control or miR-148b mimic. β-actin was loading control. Data were expressed as mean ± SD ( * P<0.05, ** P<0.01, *** P<0.001, n=3).

The potential targets of miR-148b in BCL cells were screened using the TargetScan bioinformatics prediction algorithm. Among the genes predicted to be targets of miR-148b, Bcl-w is an important anti-apoptotic protein and related to radiosensitivity. The wt 3'UTR or mut 3'UTR of Bcl-w was inserted into a reporter plasmid downstream of the luciferase gene (Figure 1A). These plasmids, miR-148b mimic, or inhibitor, were transiently co-transfected into Raji cells and SU-DHL-10 cells with the Renilla luciferase vector (pRL-TK). Dual-luciferase reporter assay indicated that miR-148b mimic or inhibitor altered the luciferase activity in the cells transfected with the plasmids containing the wt 3'UTR and the luciferase gene but not the negative control (Figure 1B, lanes 2 and 3; *p* < 0.05). Treatment with miR-148b mimic or inhibitor had no effect on the luciferase activity in the cells transfected with the plasmids containing of the mut 3'UTR and the luciferase gene (Figure 1C, lanes 2 and 3; *p* > 0.05). miR-148b mimic significantly decreased the levels of Bcl-w mRNA (Figure 1D, *p* < 0.01) and protein (Figure 1E) in the cells transfected with miR-148b mimic. These results suggest that Bcl-w is a direct target of miR-148b in BCL cells.

### miR-148b influences the viability and colony formation of BCL cells through targeting Bcl-w

**Figure 2:**
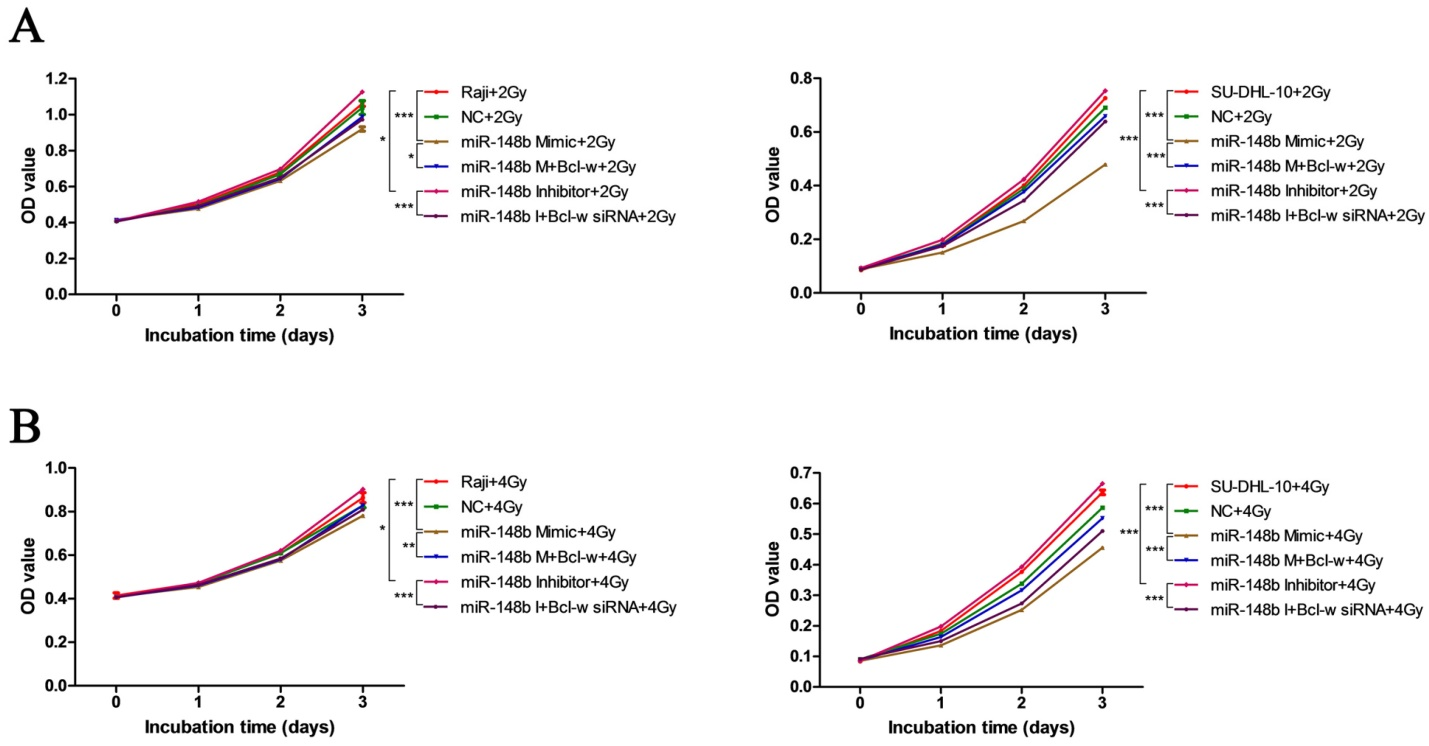
miR-148b targets Bcl-w to reduce the viability of BCL cells after irradiation. Both Raji and SU-DHL-10 cells were divided into two groups and exposed to 2 Gy or 4 Gy radiation. Cells were transfected with the same gene expression modulation reagents. The effect of these reagents on cell viability was similar at both doses. **(A)** The viability of Raji and SU-DHL-10 cells after being exposed to 2Gy and transfected with miR-148b mimic or inhibitor or Bcl-w expression vector or siRNA. **(B)** The viability of Raji and SU-DHL-10 cells after exposed to 4Gy and transfected with miR-148b mimic or inhibitor or Bcl-w expression vector or siRNA. Data were expressed as mean ± SD (* P<0.05, ** P<0.01, *** P<0.001, n=3).

**Figure 3:**
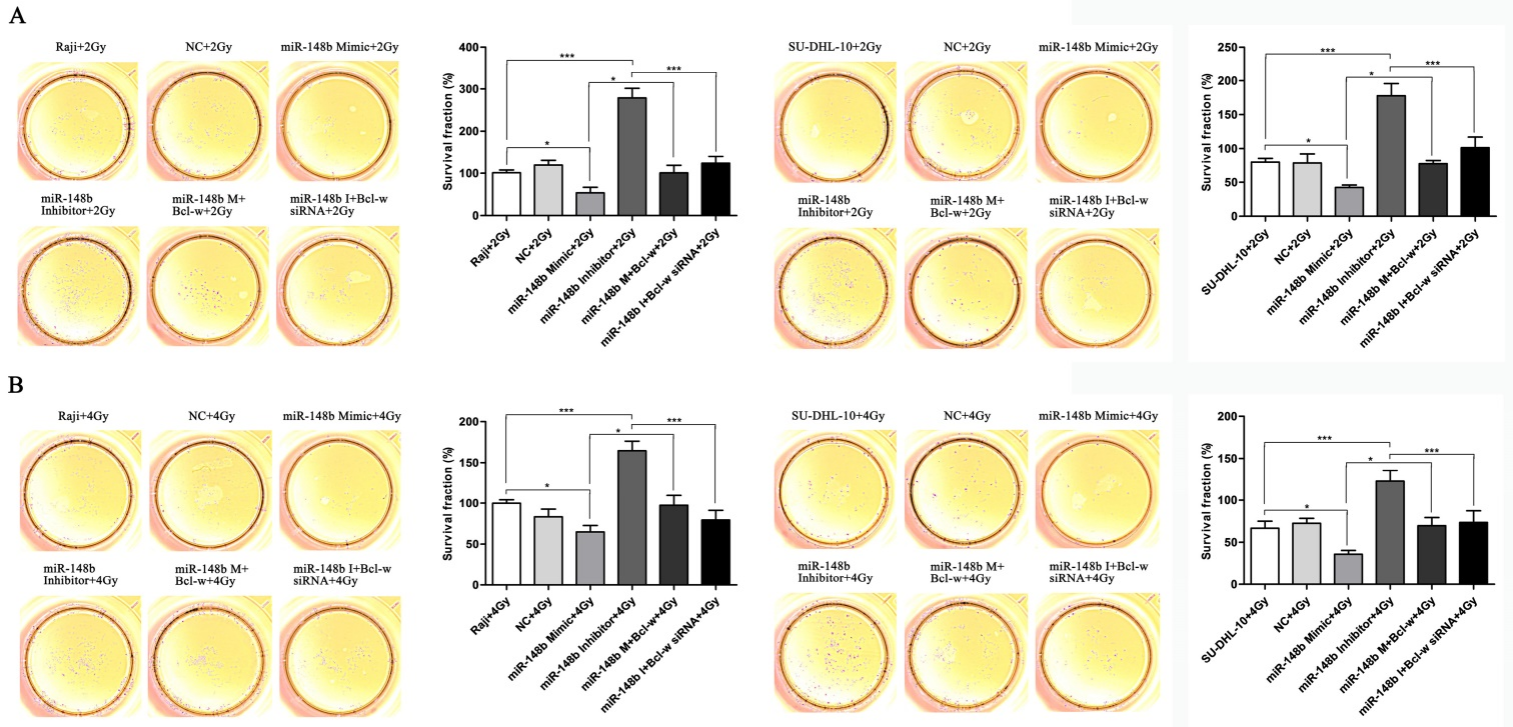
miR-148b targets Bcl-w to inhibit the colony formation of BCL cells after irradiation. Raji and SU-DHL-10 cells were divided into two groups and exposed to 2 Gy or 4 Gy radiation. Cells were transfected with the same gene expression modulation reagents. The effect of these reagents on cell colony formation was similar, regardless of the dose of radiation administered. **(A)** The colony formation of Raji cells and SU-DHL-10 cells exposed to 2Gy and transfected with miR-148b mimic or inhibitor or Bcl-w expression vector or siRNA. **(B)** The colony formation of Raji cells and SU-DHL-10 cells exposed to 4Gy and transfected with miR-148b mimic or inhibitor or Bcl-w expression vector or siRNA. Data were expressed as mean ± SD ( * P<0.05, *** P<0.001, n=3).

To investigate whether miR-148b influences the growth of BCL after ionizing radiation through Bcl-w, we investigated the effect of Bcl-w on cell viability and colony formation after ionizing radiation treatment in cells expressing various levels of miR-148b. It was found that miR-148b mimics decreased cell viability (Figure 2A-B, *p* < 0.001) and colony formation (Figure 3A-B, *p* < 0.05), while miR-148b inhibitor promoted cell viability (Figure 2A-B, *p* < 0.05) and colony formation (Figure 3A-B, *p* < 0.001), in irradiated BCL cells. Moreover, these effects of miR-148b on cell viability and colony formation were reversed by overexpression or knockdown of Bcl-w (Figure 2-3, *p* < 0.05). These results suggest that miR-148b targets Bcl-w to inhibit the viability and colony formation of BCL cells after ionizing radiation.

### miR-148b reverses Bcl-w-mediated inhibition of mitochondrial apoptosis in BCL cells

**Figure 4:**
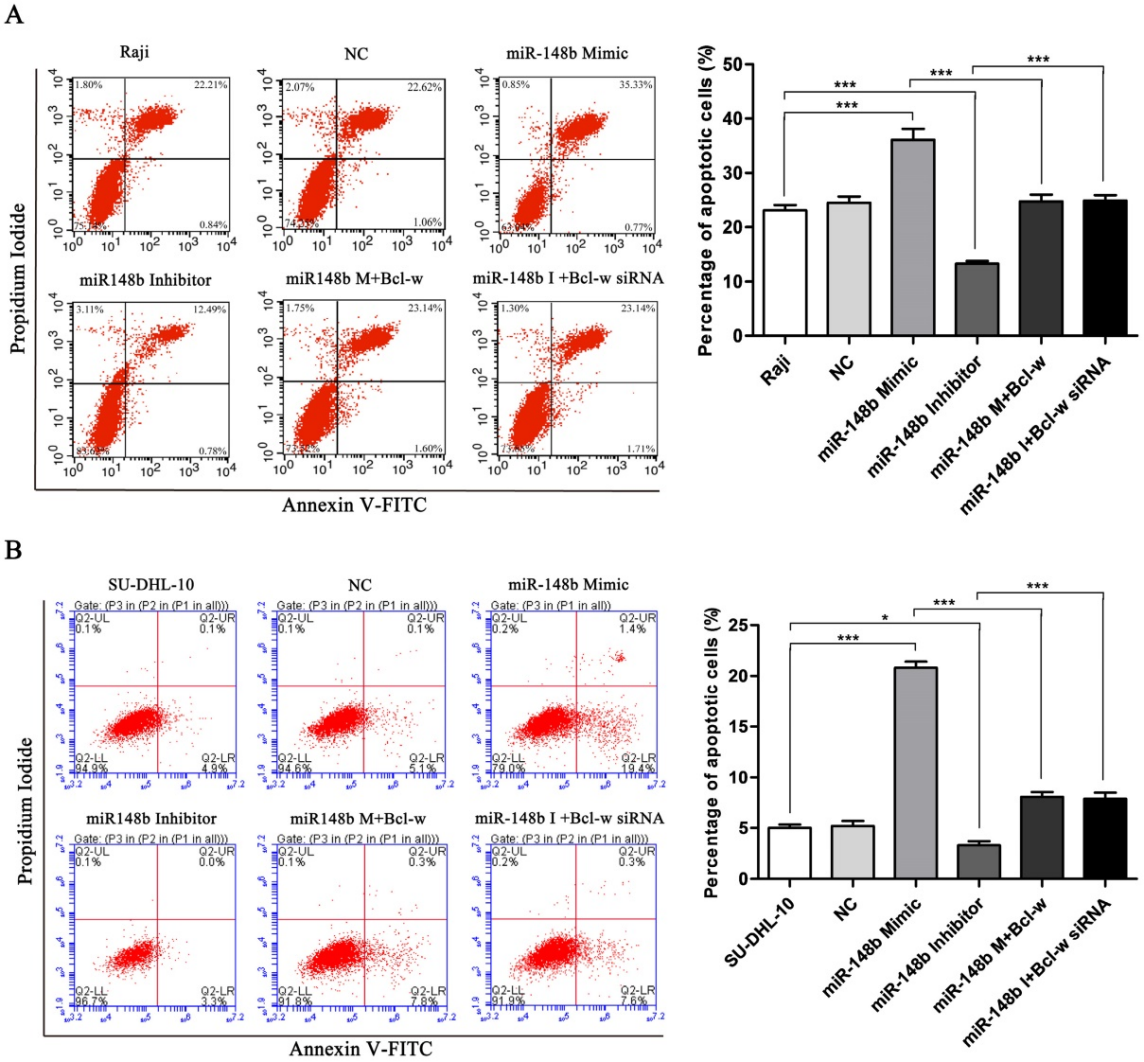
miR-148b targets Bcl-w to promote apoptosis of BCL cells after irradiation. The apoptosis of Raji **(A)** and SU-DHL-10 **(B)** cells transfected with miR-148b mimic or inhibitor or Bcl-w expression vector or siRNA. Data were expressed as mean ± SD (* P<0.05, *** P<0.001, n=3).

**Figure 5:**
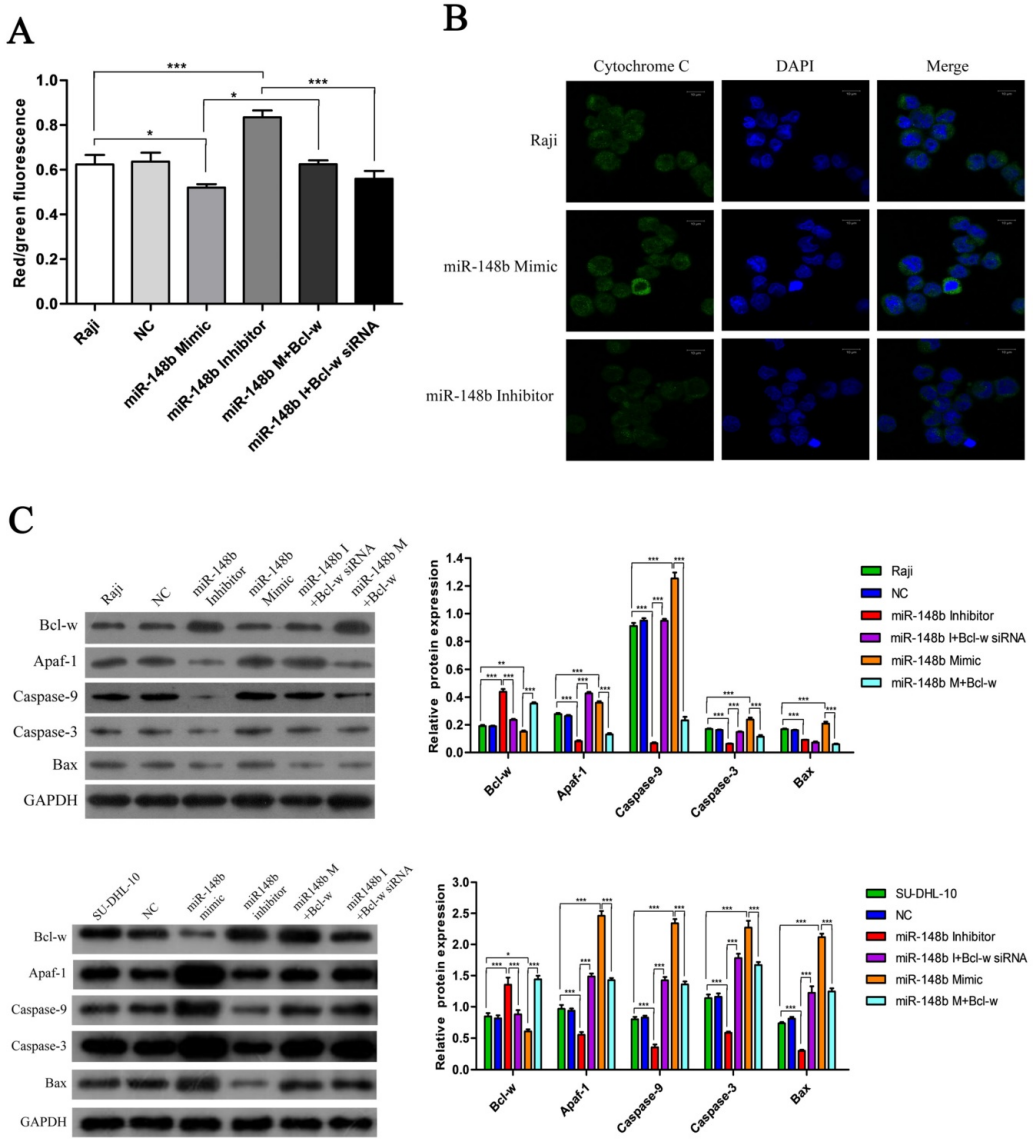
miR-148b targets Bcl-w to reverse the inhibition of mitochondrial apoptotic pathway. **(A)** The mitochondrial membrane potential of Raji cells transfected with miR-148b mimic or inhibitor or Bcl-w expression vector or siRNA. **(B)** The release of cytochrome C in Raji cells transfected with miR-148b mimic or inhibitor or Bcl-w expression vector or siRNA. **(C)** Western blot analysis of the expression of apoptosis related proteins in Raji and SU-DHL-10 cells transfected with miR-148b mimic or inhibitor or Bcl-w expression vector or siRNA. The cleaved caspase 9 and caspase 3 were quantified. GAPDH was loading control. Data were expressed as mean ± SD ( * P<0.05, ** P<0.01, *** P<0.001, n=3).

The effect of miR148b on apoptosis was examined in BCL cells with flow cytometry. It was found that the miR-148b mimic promoted apoptosis (Figure 4A-B, *p* < 0.001), while the miR-148b inhibitor inhibited apoptosis (Figure 4A-B, *p* < 0.05). It was also found that the effect of the miR-148b mimic or inhibitor could be reversed by overexpression or knockdown of Bcl-w, respectively (Figure 4A-B, *p* < 0.001). miR148b mimic decreased mitochondrial membrane potential (Figure 5A, *p* < 0.05) and increased the release of cytochrome C (Figure 5B) in Raji cells. Treatment with miR-148b inhibitor had the opposite effect (Figure 5A-B). Western blot analysis showed that treatment with miR-148b mimic activated the expression of mitochondrial apoptosis-related proteins; however, this effect was reversed by the overexpression of Bcl-w. In contrast, miR-148b inhibitor inhibited the expression of mitochondrial apoptosis-related proteins; this effect was reversed by Bcl-w knockdown (Figure 5C, *p* < 0.05). Overall, these results suggest that miR-148b targets Bcl-w to reverse the inhibition of mitochondrial apoptosis in BCL cells.

### miR-148b inhibits tumor growth in mice

**Figure 6:**
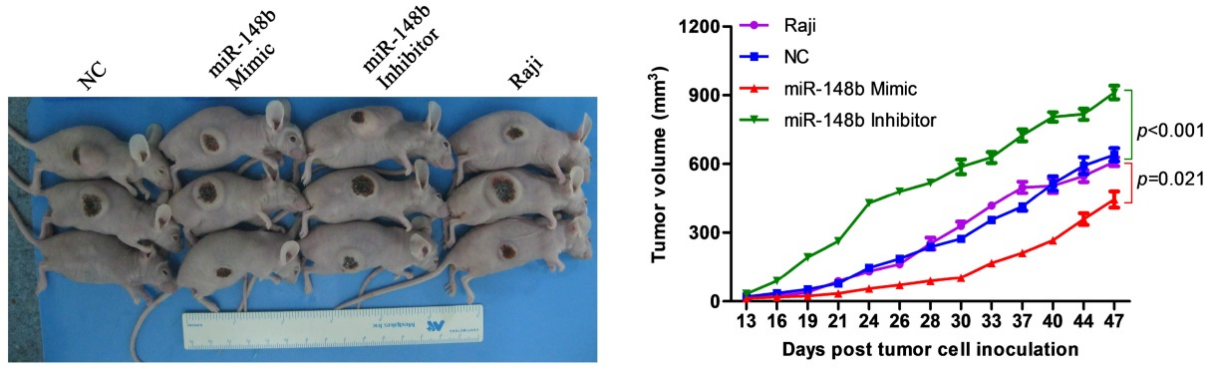
miR-148b inhibits tumor formation in irradiated Raji cells *in vivo*. Irradiated control Raji cells and irradiated Raji cells transfected with miR-148b mimic or inhibitor were inoculated subcutaneously into nude mice, tumor volume was measured and tumor growth curves were created.

Either irradiated Raji cells or irradiated Raji cells transfected with negative control siRNA (NC), miR-148b mimic, or miR-148b inhibitor were subcutaneously inoculated into the right abdomens of nude mice. The size of the tumors was measured, and a tumor growth curve was established. It was found that tumors generated from the cells transfected with the miR-148b mimic grew significantly (p=0.021) slower than the tumors generated from the cells transfected with the negative control siRNA (Figure 6). Tumors generated from the cells transfected with miR-148b inhibitor grew significantly (p < 0.001) faster than the tumors generated from the negative control cells (Figure 6).

### miR-148b is downregulated and Bcl-w is upregulated in BCL patients

**Figure 7:**
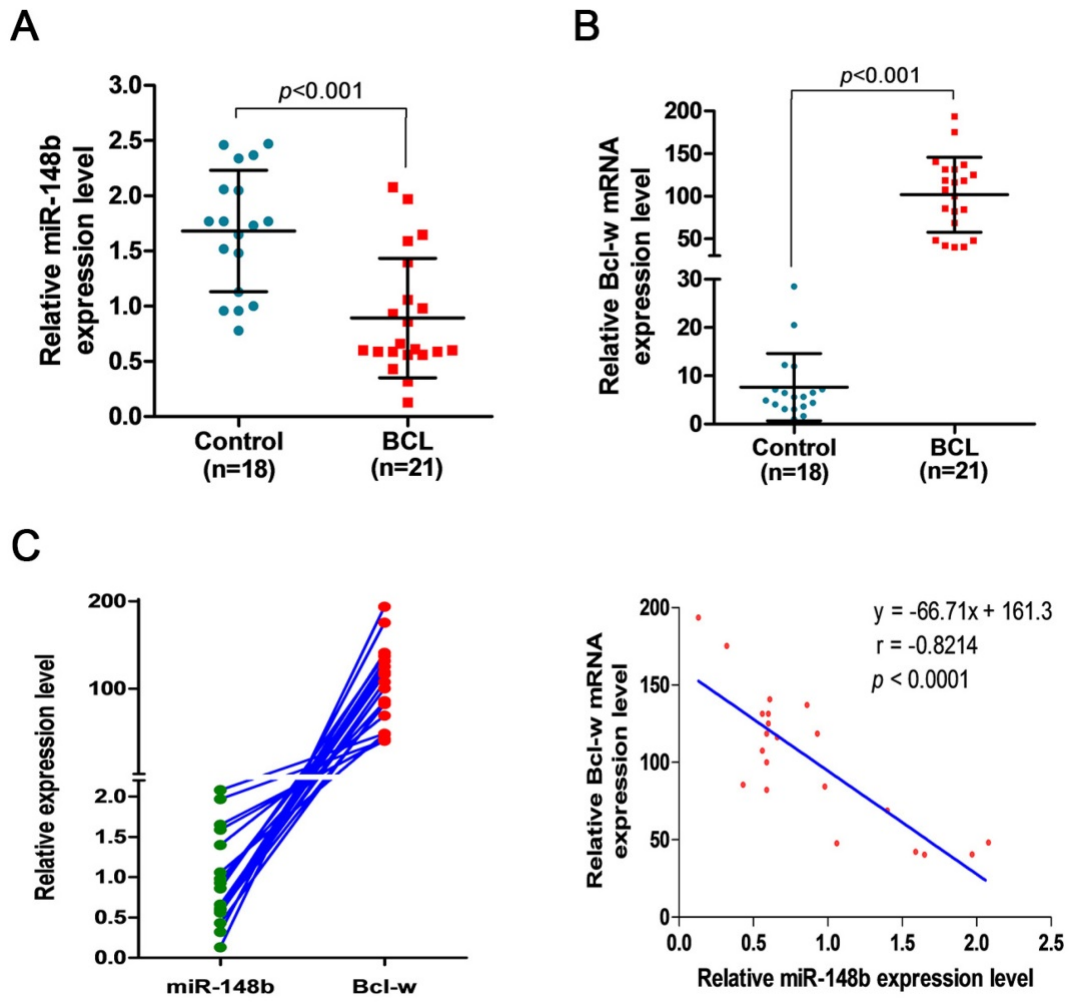
Downregulated miR-148b and upregulated Bcl-w levels in MNCs of BCL patients. **(A)** The relative expression level of miR-148b in peripheral blood MNCs was significantly lower in BCL patients than in healthy volunteers. **(B)** The relative expression level of Bcl-w in peripheral blood MNCs was significantly higher in BCL patients than in healthy volunteers.** (C)** The significant negative correlation between miR-148b and Bcl-w levels in BCL patients (γ = -0.821, P<0.001). MNC, mononuclear cell.

**Figure 8:**
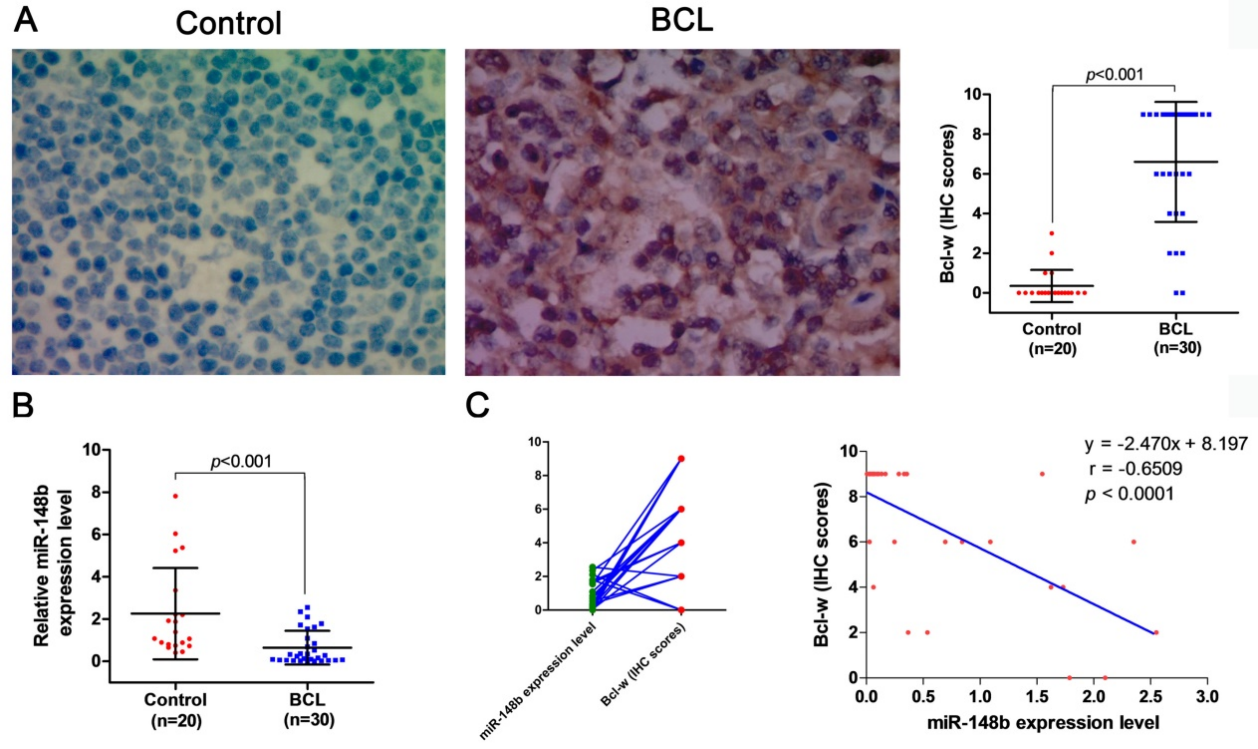
Expression levels of miR-148b and Bcl-w were detected in lymphoid tissues of BCL patients. **(A)** Assessment of differences in Bcl-w expression levels between BCL patients and normal lymphoid tissues by immunohistochemistry. **(B)** Detection of miR-148b expression levels in BCL patients and normal lymphoid tissues by qPCR. **(C)** The significant negative correlation between miR-148b and Bcl-w levels in lymphoid tissue of BCL patients (γ = -0.6509, P<0.001).

The expression levels of miR-148b were comparatively studied in the peripheral blood samples from 18 healthy volunteers and 21 BCL patients, and in lymph tissue samples from 20 healthy volunteers and 30 BCL patients. It was found that the level of miR-148b in peripheral blood mononuclear cells (MNCs) was significantly lower in BCL patients than in healthy volunteers (0.893±0.541 vs. 1.682±0.549, *p* < 0.001, Figure 7A). On the contrary, the level of Bcl-w expression in peripheral blood MNCs was significantly higher in BCL patients than in healthy volunteers (101.71±43.922 vs.7.649±6.947, *p* < 0.001, Figure 7B). The level of miR-148b was significantly negatively correlated with the level of Bcl-w levels in peripheral blood MNCs (γ = -0.821, *p* < 0.0001, Figure 7C). The level of miR-148b in lymph tissues was significantly lower in BCL patients than in healthy volunteers (0.647±0.797 vs. 2.257±2.157, *p* < 0.001, Figure 8B). On the contrary, lymph tissue Bcl-w immunohistochemistry (IHC) scores were significantly higher in BCL patients than in healthy volunteers (6.600±3.024 vs. 0.350±0.813, *p* < 0.001, Figure 8A). There was a significantly negative correlation between the level of miR148b and the Bcl-w IHC scores in lymph tissues (γ -0.651, *p* < 0.0001, Figure 8C).

### Decreased lymph tissue cell apoptosis is associated with the expression of miR-148b and Bcl-w in BCL patients

**Figure 9:**
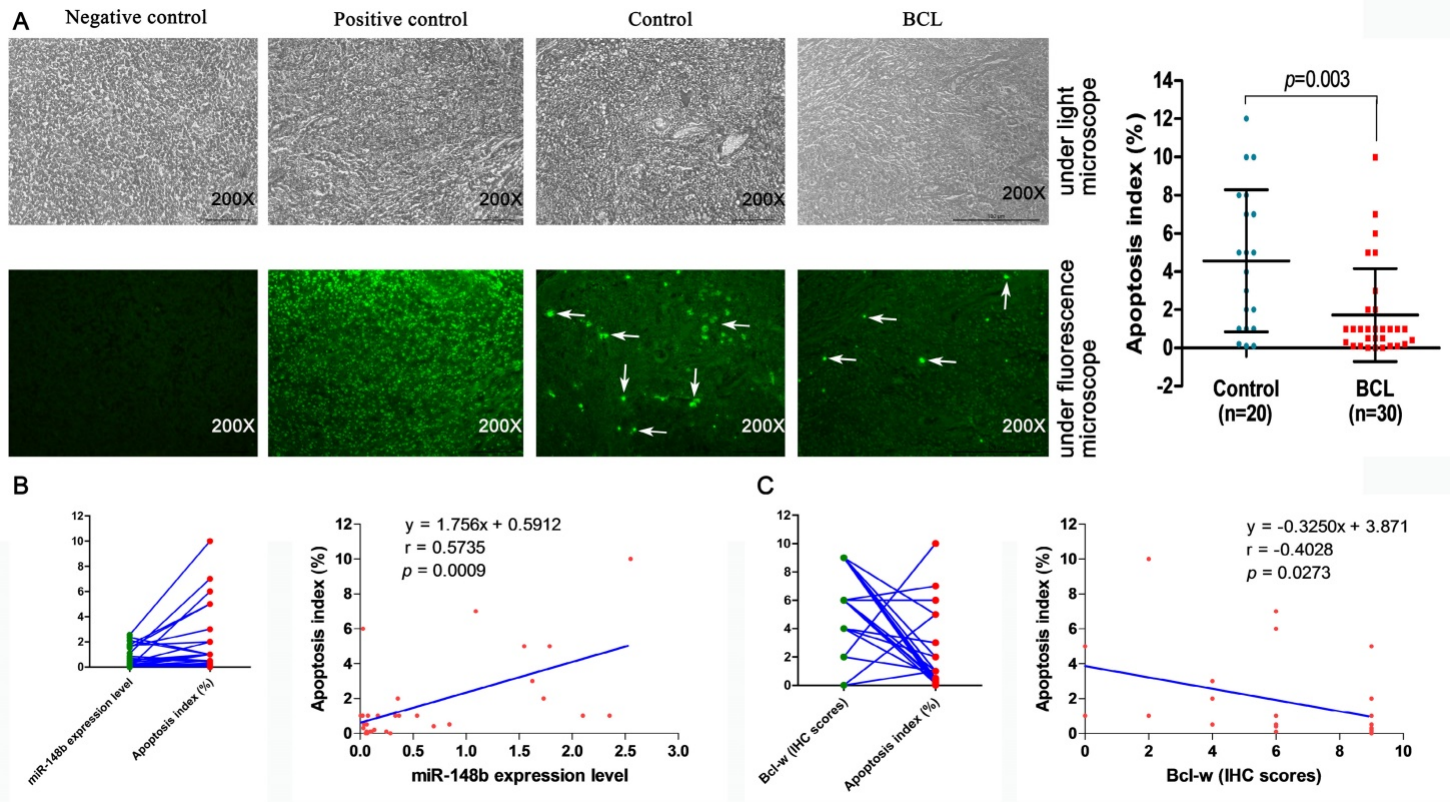
Correlation between expression levels of miR-148b and Bcl-w and apoptosis in lymphoid tissues of patients with BCL. **(A)** Detection of apoptosis in BCL patients and normal lymphoid tissues by TUNEL assay. **(B)** Relationship between expression level of miR-148b and apoptosis in lymphoid tissues. **(C)** Relationship between IHC score of Bcl-w and apoptosis in lymphoid tissues. IHC, immunohistochemistry.

The relationship between cell apoptosis and miR-148b was studied in the lymphatic tissues of 20 healthy volunteers and 30 BCL patients by TUNEL assay. The lymphatic tissue apoptosis index was significantly lower in BCL patients than in healthy volunteers (1.727±2.440 vs. 4.570±3.723, p = 0.003, Figure 9A). The apoptosis index was significantly correlated with the level of miR-148b in the lymphatic tissue of BCL patients (γ = 0.5735, *p* = 0.0009, Figure 9B). On the contrary, there was a negative correlation between apoptosis index and Bcl-w IHC scores (γ = -0.4028, *p* = 0.0273, Figure 9C).

## Discussion

In this study, it was found that miR-148b targeted Bcl-w in BCL cells, influenced the number of viable cells and colony formation through targeting Bcl-w and reversed Bcl-w-mediated inhibition of mitochondrial apoptosis in irradiated BCL cells, and inhibited the growth of the tumors generated from the xenograft BCL cells in mice. In BCL patients, miR-148b was downregulated and Bcl-w is upregulated in BCL patients; miR-148b was negatively correlated with Bcl-w in both peripheral blood MNCs and lymph tissues; cell apoptosis was decreased in the lymph tissues, which was associated with the expression of miR-148b and Bcl-w. These results demonstrated that miR-148b increased the radiosensitivity of B-cell lymphoma cells by targeting Bcl-w to promote apoptosis.

The dual-luciferase reporter assay indicated that miRNA-148b directly targeted the 3'UTR of Bcl-w and suppressed the expression of Bcl-w mRNA and protein in cultured BCL cells, which confirms the proposal in a previous study that Bcl-w may be a target of miRNA-148b 35. The downregulation of miR-148b, upregulation of Bcl-w and the negative correlation between miR-148b level and Bcl-w level in the peripheral blood and lymph tissues of BCL patients suggest that miR-148b suppresses the expression of Bcl-w *in vivo*.

Our study showed that miR-148b mimics decreased cell viability and inhibited colony formation while miR-148b inhibitor promoted cell viability and colony formation in irradiated BCL cells. These effects of miR-148b on cell viability and colony formation were reversed by overexpression or knockdown of Bcl-w. In irradiated BCL cell xenograft mice miR-148b inhibited the tumor growth while miR-148b inhibitor promoted the tumor growth. These results suggest that miR-148b targets Bcl-w to decrease the number of viable cells so as to inhibit the tumor growth. miR-148b mimic triggered an increase in mitochondrial mediated apoptosis while miR-148b inhibitor decreased mitochondrial mediated apoptosis in BCL cells, suggesting that miR-148b promotes mitochondrial mediated apoptosis in BCL cells, which is in agreement with our previous observation 33. The reversion of miR-148b mimic and inhibitor mediated effects on mitochondrial mediated apoptosis by intervention of Bcl-w expression indicated that miR-148b increased mitochondrial mediated apoptosis through targeting Bcl-w, which led to the decrease in the number of viable cells and reduction in tumor growth in irradiated BCL cells.

It was found that in BCL patients, miR-148b was downregulated and Bcl-w is upregulated; miR-148b was negatively correlated with Bcl-w in both peripheral blood MNCs and lymph tissues; cell apoptosis was decreased in the lymph tissues; cell apoptosis index was positively correlated with the expression of miR-148b and negatively correlated with Bcl-w. Based on the observation in this study that miR-148b directly targeted Bcl-w, it is obvious that miR-148b increases apoptosis by suppressing Bcl-w in BCL patients.

Apoptosis is essential for maintaining tissue balance and cellular integrity 39. Malignant tumors may develop when apoptosis-related genetic mutations or abnormal expression block apoptosis; tumor treatment may induce apoptosis-related gene expression to promote tumor cell apoptosis 40-44. Previously we found that miR-148b, which promotes radiation-induced apoptosis, enhanced the radiosensitivity of BCL cells 33. In this study, it was found that miR-148b inhibited tumor growth by targeting Bcl-w and promoting apoptosis in irradiated BCL cells, and promoted apoptosis by suppressing Bcl-w in BCL patients. These observations indicate that miR-148b increases radiosensitivity of BCL cells by targeting Bcl-w to increase apoptosis.

## Conclusion

In conclusion, miR-148b directly targets Bcl-w and regulates the expression of Bcl-w in BCL patients and cultured BCL cells. miR-148b increased the apoptosis in BCL patients, decreased the number of viable cells, colony formation and tumor growth through increasing apoptosis in irradiated BCL cells. miR-148b promotes radiosensitivity by targeting Bcl-w to activate mitochondrial mediated apoptosis in BCL cells.

## Figures and Tables

**Table 1:** Clinical information of BCL and healthy volunteers

Variables	Peripheral blood	Lymphatic tissue	*p* value
Age (mean±SD, years)	57±18	59±15	0.668^*^
Gender, n (%)			0.517^#^
Male	12 (57.1)	21 (70.0)	
Female	9 (42.9)	9 (30.0)	
Histology, n (%)			0.128^#^
Diffuse large B-cell lymphoma	9 (42.9)	13 (43.3)	
Marginal zone lymphoma	6 (28.6)	3 (10.0)	
Follicular lymphoma	5 (23.8)	5 (16.7)	
Mantle cell lymphoma	1 (4.7)	4 (13.3)	
Burkitt lymphoma	0 (0.0)	5 (16.7)	
Stage, n (%)			0.484^#^
I	1 (4.8)	4 (13.3)	
II	2 (9.5)	6 (20.0)	
III	11 (52.4)	13 (43.3)	
IV	7 (33.3)	7 (23.4)	
Healthy volunteers, n	18	20	

Note: ^*^ Independent-sample Student 's t-test; ^#^ Chi-square test.

**Table 2:** Sequences of the primers

Target	Sequence 5' - 3'
Bcl-w wt (F)	CCGCTCGAGAAGTCCAGGGCCAGGTGGG
Bcl-w wt (R)	ATAAGAATGCGGCCGCTCAGTCCTTCTCATTAAACTTCTGGG
Bcl-w mut(F)	AACCCTGCCTGTGGTCCTGACGTGACTTCACCTTAGCTAGACCATGG
Bcl-w mut(R)	CCATGGTCTAGCTAAGGTGAAGTCACGTCAGGACCACAGGCAGGGTT
miR-148b (F)	CCGCTCGAGTCATTTGCAGCAGCCTAGTTGC
miR-148b (R)	CGCGGATCCACTGAGAAATGGGCTTCCAGGAC
miR-148b inhibitor (F)	CGCGGATCCCCGGACAAAGTTCTTCATGCAC
miR-148b inhibitor (R)	CCGGAATTCCCGGTCAGTGCATGAAGAACTT
ORF of Bcl-w (F)	CGGGGTACCGCCACCATGGCGACCCCAGCCTCGGCCCC
ORF of Bcl-w (R)	CCGCTCGAGTCACTTGCTAGCAAAAAAGGCCCCTAC
has-miR-148b-3p- RT	GTCGTATCCAGTGCAGGGTCCGAGGTATTCGCACTGGATACGACCACAAAGTT
has-mir-148b (F)	ATGGTTCGTGGGTCAGTGCATCACAGAACTTT
has-mir-148b (R)	GTGCAGGGTCCGAGGT
U6 (F)	CTCGCTTCGGCAGCACA
U6 (R)	AACGCTTCACGAATTTGCGT
U6-RT	GTCGTATCCAGTGCAGGGTCCGAGGTATTCGCACTGGATACGACCAAATATGGAAC
Bcl-w (F)	CTTCACCCAGGTCTCCGATG
Bcl-w (R)	CCCACCAGTGGTTCCATCTC
β-actin (F)	CATGTACGTTGCTATCCAGGC
β-actin (R)	CTCCTTAATGTCACGCACGAT

## Abbreviations

miR-148b: MicroRNA-148b
Bcl-w: B cell lymphoma-w
BCL: B-cell lymphoma
MNC: mononuclear cell
wt 3'-UTR: wild-type 3'-Untranslated region
mut 3'-UTR: mutated 3'-Untranslated region
GFP: Green fluorescent protein
siRNA: small interfering RNA
qRT-PCR: Real-time quantitative polymerase chain reaction
IHC: Immunohistochemistry
TUNEL: Terminal deoxynucleotidyl transferase-mediated dUTP nick-end labeling
CCK-8: Cell Counting Kit-8
MMP: Mitochondrial membrane potential
Annexin V/PI: Annexin V / propidium iodide
